# Comparison of measured Varian Clinac 21EX and TrueBeam accelerator electron field characteristics

**DOI:** 10.1120/jacmp.v16i4.5496

**Published:** 2015-07-08

**Authors:** Samantha A.M. Lloyd, Sergei Zavgorodni, Isabelle M. Gagne

**Affiliations:** ^1^ Department of Physics and Astronomy University of Victoria Victoria BC Canada; ^2^ Department of Medical Physics British Columbia Cancer Agency – Vancouver Island Centre Victoria BC Canada

**Keywords:** TrueBeam, electron therapy, modulated electron radiation therapy

## Abstract

Dosimetric comparisons of radiation fields produced by Varian's newest linear accelerator, the TrueBeam, with those produced by older Varian accelerators are of interest from both practical and research standpoints. While photon fields have been compared in the literature, similar comparisons of electron fields have not yet been reported. In this work, electron fields produced by the TrueBeam are compared with those produced by Varian's Clinac 21EX accelerator. Diode measurements were taken of fields shaped with electron applicators and delivered at 100 cm SSD, as well as those shaped with photon MLCs without applicators and delivered at 70 cm SSD for field sizes ranging from 5×5 to $25\times 25\;{\text{cm}}^{2}$ at energies between 6 and 20 MeV. Additionally, EBT2 and EBT3 radio‐chromic film measurements were taken of an MLC‐shaped aperture with closed leaf pairs delivered at 70 cm SSD using 6 and 20 MeV electrons. The 6 MeV fields produced by the TrueBeam and Clinac 21EX were found to be almost indistinguishable. At higher energies, TrueBeam fields shaped by electron applicators were generally flatter and had less photon contamination compared to the Clinac 21EX. Differences in PDDs and profiles fell within 3% and 3 mm for the majority of measurements. The most notable differences for open fields occurred in the profile shoulders for the largest applicator field sizes. In these cases, the TrueBeam and Clinac 21EX data differed by as much as 8%. Our data indicate that an accurate electron beam model of the Clinac 21EX could be used as a starting point to simulate electron fields that are dosimetrically equivalent to those produced by the TrueBeam. Given that the Clinac 21EX shares head geometry with Varian's iX, Trilogy, and Novalis TX accelerators, our findings should also be applicable to these machines.

PACS number: 87.56.bd

## I. INTRODUCTION

Varian's most recent generation of clinical linear accelerator, the TrueBeam (Varian Medical Systems, Palo Alto, CA), is a rebuild “from the ground up.” As a result of the reengineering process, there are differences in the design of the accelerator head compared to older Varian machines, and although the internal specifications of the TrueBeam are proprietary, some of the changes are known qualitatively. In particular, the bending magnet, carrousel, and electron scattering foils have been redesigned, the primary collimator is thicker than in older units, and an antibackscatter foil has been added.^1^, ^2^, ^3^

Over the last few years, a number of articles have been published characterizing the dosimetric properties of the TrueBeam, comparing multiple TrueBeams with one another, and comparing the TrueBeam with older Varian accelerators. Chang et al.,^(1)^ Beyer,^(2)^ and Glide‐Hurst et al.^(3)^ performed comparisons of select photon and electron fields from unmatched TrueBeam units; in the Beyer and Glide‐Hurst et al. studies, the units were installed in different centers. All three studies found their respective machines to be in excellent agreement with one another, Beyer citing percent depth dose (PDD), profile, and output measurements to be reproducible between machines within 2%. Overall, the TrueBeam was found to be dosimetrically consistent from one machine to the next.

Additionally, Beyer compared TrueBeam photon fields with those produced by Varian Trilogy and Clinac 2100 accelerators to evaluate the possibility of beam‐matching, and showed output factors to agree within 2%, PDDs to agree within 1%, and profiles to agree within 2% and 1 mm, for the most part, between the three machines. Slight differences in profile and penumbra width were attributed to changes in the material and design of the flattening filter and other head components that contribute to scatter, as well as to changes in the bending magnet impacting the incident electron source width. This was also cited as the cause of differences in profile shoulder shape at distances greater than 20 cm away from the central axis. In general, TrueBeam photon fields were found to be similar to those produced by previous Varian units, with differences occurring for small fields and very large fields.

To the best of our knowledge, a dosimetric comparison of electron fields produced by the TrueBeam and those produced by older Varian accelerators has not been presented in the literature. In addition to evaluating the differences in beam quality between Varian machines, such a comparison would be useful for the development of Monte Carlo electron beam models used for accurate dose calculations. While Varian has recently released phase‐space source files for each of the electron energies available on the TrueBeam,^(4)^ such models are useful for continued research into treatment techniques involving modulated electron fields (MERT).^(5)^

The purpose of this work, therefore, is to present a dosimetric comparison of electron fields generated by the TrueBeam with those generated by an older Varian accelerator, the Clinac 21EX. Specifically, this study is concerned with fields ranging in size from 5×5 to $25\times 25\;{\text{cm}}^{2}$ and in energy from 6 to 20 MeV, shaped using electron applicators at 100 cm source to surface distance (SSD), and by multileaf collimators (MLCs) without applicators at a short SSD as would be employed in MERT. The accelerators used in this work were not matched, and no attempt was made to match them. These comparisons will aid in evaluating the potential use of existing Monte Carlo models of the Clinac 21EX as a starting point to simulate electron fields that are dosimetrically equivalent to those generated by the TrueBeam. As well, given that the Clinac 21EX head geometry is shared by Varian's iX, Trilogy, and Novalis TX accelerators, it is expected that this comparison can be extended to the latter machines.

## II. MATERIALS AND METHODS

### A. Diode measurements

#### A.1 Applicator‐defined apertures

An initial comparison of the Clinac 21EX and the TrueBeam was performed using commissioning measurements acquired following the installation of each accelerator. These measurements included depth and profile scans taken at 100 cm SSD with 6×6,10×10,20×20, and $25\times 25\;{\text{cm}}^{2}$ electron applicators, using 6, 9, 12, 16, and 20 MeV electrons. Default jaw settings were used for each of the applicator sizes as programmed for each machine. Based on the schematics provided in Varian's Monte Carlo Data Package for the Clinac 21EX and the limited schematics made available in the TrueBeam Monte Carlo Data Package for patient‐dependent beam modifiers, electron applicators are the same for both machines. Commissioning data for the Clinac 21EX was collected using diodes as the field and reference detectors and processed using OmniPro‐Accept v6.6 (IBA Dosimetry, formerly Scanditronix/Wellhofer, Schwarzenbruck, Germany). TrueBeam measurements were performed using an electron diode as the field detector and CC13 ion chamber as reference, and processed using OmniPro‐Accept v7.4 (IBA Dosimetry). Both sets of measurements were performed in a large water tank ($48\times 48\times 48\;{\text{cm}}^{3}$) with profile measurements taken at $D_{\text{max}}$.

#### A.2 MLC‐defined apertures

Measurements of MLC‐defined apertures were performed at short SSD to minimize electron scatter. Electron applicators were not used for these measurements and delivery was performed in service mode for the Clinac 21EX and in research mode for the TrueBeam. Both accelerators are equipped with the Varian Millenium 120‐leaf MLC with 40 central leaf pairs and 20 outer leaf pairs whose widths project to 0.5 and 1.0 cm at isocenter, respectively. The same water tank was used to take depth and profile scans at 70 cm SSD for 5×5 and $20\times 20\;{\text{cm}}^{2}$ apertures with secondary collimating jaws set to 6×6 and $21\times 21\;{\text{cm}}^{2}$, respectively, using 6, 12, and 20 MeV electrons. An electron diode and CC13 ion chamber reference were used to perform measurements on both the Clinac 21EX and TrueBeam accelerators. MLC‐shaped aperture data were acquired at the end of the Clinac 21EX accelerator's clinical life and within the first year of the TrueBeam's installation.

Profiles were acquired at nominal values of $d_{\text{max}}$ where $d_{\text{max}}=1.4,2.7$, and 2.2 cm for 6, 12, and 20 MeV, respectively. All data was processed using OmniPro‐Accept v7.4.

### B. Film measurements

**Figure 1:**
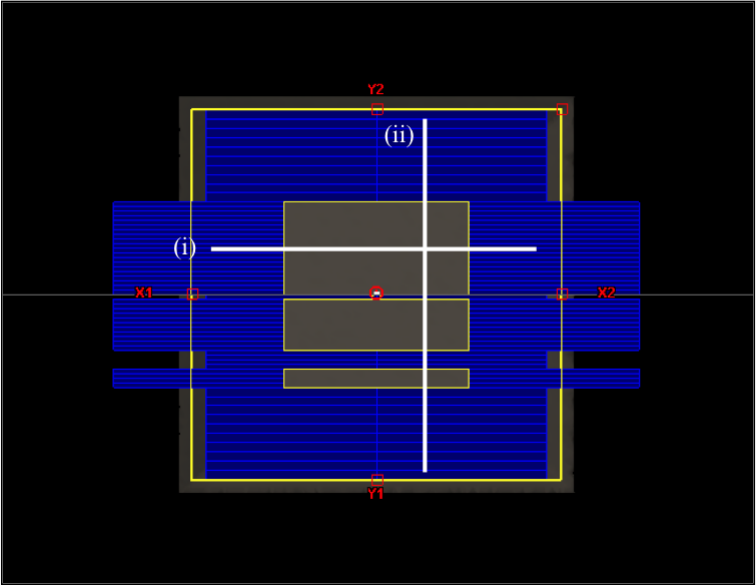
Beam's eye view of the MLC aperture used to deliver 6 and 20 MeV electrons to film in solid water at 70 cm SSD. Crossline and inline profiles were extracted along the lines (i) and (ii), respectively.

Film measurements of the MLC‐aperture shown in Fig. 1 were performed using Gafchromic EBT2 (Clinac 21EX) and EBT3 (TrueBeam) radio‐chromic film (Ashland Inc., Covington, KY) in order to compare in‐field leaf‐edge effects on dose. The use of EBT3 for the TrueBeam was due to the transition from EBT2 to EBT3 during the timeframe of these measurements. Both EBT2 and EBT3 film have been found to be suitable for electron dosimetry in studies by Arjomandy et al.^(6)^ and Chan et al.,^(7)^ respectively. Reinhardt et al.^(8)^ compared the performance of the films and concluded that EBT2 and EBT3 have similar dosimetric performances with the elimination of side orientation dependencies and the reduction of Newton's rings in the case of EBT3.

The MLC aperture used was based on a $20\times 20\;{\text{cm}}^{2}$ open field with five closed leaf pairs. Fields were delivered at 70 cm SSD with film placed between slabs of Solid Water (Gammex Inc., Middleton, WI) perpendicular to the beam axis at depths of 1.5 and 2.0 cm for 6 and 20 MeV electrons, respectively. Irradiated films were scanned on an Epson Expression 10000XL scanner (Seiko Epson Corporation, Suwa, Japan) in transmission mode at 150 dpi and processed using MATLAB (Mathworks, Natick, MA), according to the procedure outlined by Garcia and Azorin.^(9)^ Using the red channel from each scan, images were corrected for scanner and film nonuniformities by subtracting a scan of an unirradiated film. The resulting images were smoothed using a 2D median filter (∼1×1 mm) and converted to optical density (OD). Conversion from OD to dose was done using calibration curves specific to each dataset. These calibration curves were constructed by irradiating small pieces of film to known doses between 0 and 300 cGy in 50 cGy increments and fitting their responses to a second order polynomial. To assess film dose‐measurement uncertainty, four different films irradiated with the same field and energy were scanned and processed, and the standard deviation between each pixel value was evaluated.

## III. RESULTS

### A. Diode measurements

#### A.1 Applicator‐defined apertures

Relative dose measurements of applicator‐shaped apertures delivered at 100 cm SSD are plotted in 2, 3, which show depth and crossline profile data, respectively. A gamma comparison of Clinac 21EX and TrueBeam depth measurements show that differences between nearly all data points fall within 2% and 2 mm. Similar comparisons of profile data show the same agreement for 6×6 and $10\times 10\;{\text{cm}}^{2}$ applicators at all energies, but there is drop in gamma pass rates for the larger field sizes at 9 and 12 MeV. Visually, this is corroborated by obvious differences in flatness for 20×20 and $25\times 25\;{\text{cm}}^{2}$ applicators at energies of 9 MeV and greater.

Table 1 summarizes depth and profile characteristics for the fields plotted in 2, 3, including $d_{80},d_{50}$, FWHM, 80%−20% penumbra width, photon contamination ($D_{x}$) and field flatness (the variation over the mean within 80% of the FWHM). All but eight (of 120) parameter comparisons agree within 2% or 2 mm, and all agree within 3% or 3 mm. The machines differ in field flatness by as much as 2.5% and there is an overall reduction in photon contamination in fields produced by the TrueBeam.

**Figure 2:**
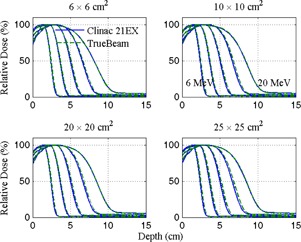
Diode measured depth doses for 6, 9, 12, 16, and 20 MeV electrons shaped by 6×6,10×10,20×20, and $25\times 25\;{\text{cm}}^{2}$ applicators delivered at 100 cm SSD. Data are normalized at $d_{\text{max}}$ along the central axis.

**Figure 3:**
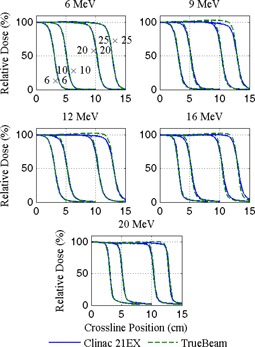
Diode measured crossline dose profiles at $d_{\text{max}}$ for 6, 9, 12, 16, and 20 MeV electrons shaped by 5×5,10×10,20×20, and $25\times 25\;{\text{cm}}^{2}$ applicators delivered at 100 cm SSD. Data are normalized at $d_{\text{max}}$ along the central axis.

**Table 1:** Depth and profile characteristics for 6, 9, 12, 16, and 20 MeV electrons shaped by 6×6,10×10,20×20, and $25\times 25\;{\text{cm}}^{2}$ applicators delivered at 100 cm SSD

*Applicator Size (cm^2^)*	6×6		10×10		20×20		25×25	
	*21EX*	*TB*	*21EX*	*TB*	*21EX*	*TB*	*21EX*	*TB*
*6 MeV*								
$d_{80}$ (cm)	2.0	2.1	2.0	2.1	2.0	2.1	2.0	2.1
$d_{50}$ (cm)	2.4	2.5	2.4	2.5	2.4	2.5	2.4	2.5
FWHM (cm)	6.0	6.1	10.2	10.2	20.4	20.1	25.6	25.6
80%−20% (cm)	1.1	1.1	1.1	1.1	1.2	1.1	1.1	1.1
$D_{x}$ (%)	0.1	0.2	0.5	0.3	0.4	0.3	0.4	0.4
Flatness (%)	10.6	10.0	4.1	4.2	1.4	1.4	1.2	1.1
*9 MeV*								
$d_{80}$ (cm)	3.1	3.1	3.1	3.2	3.1	3.2	3.1	3.2
$d_{50}$ (cm)	3.6	3.7	3.6	3.7	3.7	3.7	3.6	3.7
FWHM (cm)	6.2	6.1	10.3	10.3	20.7	20.7	25.8	25.8
80%−20% (cm)	1.2	1.0	1.2	1.0	1.2	1.0	1.3	1.0
$D_{x}$ (%)	0.4	0.4	0.9	0.4	0.6	0.5	0.8	0.6
Flatness (%)	10.3	8.7	4.4	2.5	0.9	1.0	1.6	1.5
*12 MeV*								
$d_{80}$ (cm)	4.3	4.3	4.3	4.4	4.3	4.4	4.3	4.4
$d_{50}$ (cm)	5.0	5.1	5.0	5.1	5.0	5.2	5.0	5.2
FWHM (cm)	6.2	6.2	10.4	10.4	20.8	20.9	26.1	26.4
80%−20% (cm)	1.2	1.2	1.2	1.2	1.3	1.1	1.4	1.1
$D_{x}$ (%)	0.8	0.5	1.2	0.6	1.4	0.7	1.4	0.9
Flatness (%)	10.6	10.2	6.0	4.0	0.9	0.9	1.3	2.0
*16 MeV*								
$d_{80}$ (cm)	5.4	5.5	5.6	5.8	5.7	5.8	5.6	5.8
$d_{50}$ (cm)	6.6	6.7	6.6	6.7	6.7	6.8	6.6	6.8
FWHM (cm)	6.2	6.2	10.5	10.4	21.0	20.9	26.2	26.1
80%−20% (cm)	0.8	0.9	1.2	0.9	1.2	0.9	1.2	0.9
$D_{x}$ (%)	1.6	1.0	2.3	1.2	2.5	1.4	2.4	1.7
Flatness (%)	5.1	6.8	4.5	2.0	0.4	0.8	0.8	1.2
*20 MeV*								
$d_{80}$ (cm)	6.4	6.5	6.9	6.9	7.0	7.0	7.0	7.0
$d_{50}$ (cm)	8.1	8.1	8.4	8.4	8.4	8.5	8.4	8.5
FWHM (cm)	6.2	6.2	10.4	10.4	20.8	20.7	25.8	25.9
80%−20% (cm)	0.6	0.6	0.8	0.6	0.8	0.6	0.6	0.6
$D_{x}$ (%)	3.0	1.8	4.0	2.1	4.4	2.4	4.0	3.0
Flatness (%)	2.4	2.9	1.9	1.1	1.9	0.7	1.3	1.2

$d_{80}=\text{depth}$ at 80% dose; $d_{50}=\text{depth}$ at 50% dose; FWHM=full width at half maximum; 80%−20%=average width of the penumbra measured between 80% and 20% doses; $D_{x}=\text{dose beyond}$ the electron range due to photon contamination; Flatness=variation over the mean within 80% of the FWHM.

#### A.2 MLC‐defined apertures

**Figure 4:**
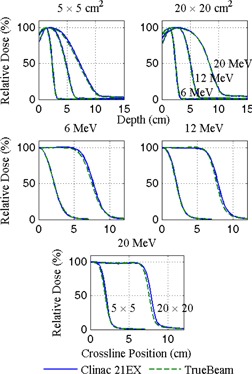
Diode measured depth and crossline profile curves for 6, 12, and 20 MeV electrons delivered at 70 cm SSD with 5×5 and $20\times 20\;{\text{cm}}^{2}$ MLC‐shaped apertures. Data are normalized at $d_{\text{max}}$ along the central axis.

Figure 4 shows depth and crossline profile measurements taken at 70 cm SSD for 6, 12, and 20 MeV electrons with 5×5 and $20\times 20\;{\text{cm}}^{2}$ MLC‐shaped apertures and jaws set to 6×6 and $21\times 21\;{\text{cm}}^{2}$, respectively. Again, differences in depth measurements fall within 2% and 2 mm. Profile differences for $5\times 5\;{\text{cm}}^{2}$ apertures fall within 2% and 2 mm for all energies while profiles measured with $20\times 20\;{\text{cm}}^{2}$ apertures require that the distance to agreement be extended to 3 mm.

Table 2 summarizes depth and profile characteristics for the MLC‐shaped electron fields plotted in Fig. 4. For the $5\times 5\;{\text{cm}}^{2}$ field, all but one parameter agree within 2% or 2 mm, and the outlier agrees within 3 mm. At $20\times 20\;{\text{cm}}^{2}$, all tabulated parameters agree within 2% or 2 mm with the exception of FWHM, which differ by as much as 5 mm. Note, however, that the difference in width represents twice the distance to agreement. Compared to applicator‐defined fields, MLC field definition produces poorer field flatness, overall, while the difference in the flatness between machines is far less pronounced, within 1.7%.

**Table 2:** Depth and profile characteristics for 6, 12, and 20 MeV electrons delivered at 70 cm SSD shaped by $5\times 5\;{\text{cm}}^{2}$ and $20\times 20\;{\text{cm}}^{2}$ MLC apertures

*Aperture Size (cm^2^)*	5×5		20×20	
	*21EX*	*TB*	*21EX*	*TB*
*6 MeV*				
$d_{80}$ (cm)	2.0	2.0	2.0	2.1
$d_{50}$ (cm)	2.4	2.4	2.4	2.5
FWHM (cm)	4.4	4.3	15.4	15.0
80%−20% (cm)	1.8	1.9	2.0	2.2
$D_{x}$ (%)	0.1	0.2	0.1	0.1
Flatness (%)	20.2	20.3	5.5	6.2
*12 MeV*				
$d_{80}$ (cm)	3.8	3.7	4.3	4.3
$d_{50}$ (cm)	4.9	4.8	5.1	5.0
FWHM (cm)	4.2	4.1	15.8	15.4
80%−20% (cm)	1.4	1.5	1.8	1.6
$D_{x}$ (%)	0.2	0.2	0.4	0.3
Flatness (%)	17.9	18.5	3.6	2.7
*20 MeV*				
$d_{80}$ (cm)	5.4	5.1	6.8	6.8
$d_{50}$ (cm)	7.3	7.1	8.4	8.4
FWHM (cm)	4.0	3.8	15.6	15.1
80%−20% (cm)	0.8	0.9	1.0	1.0
$D_{x}$ (%)	0.9	1.3	1.8	0.9
Flatness (%)	11.1	12.8	1.2	0.9

### B. Film measurements

**Figure 5:**
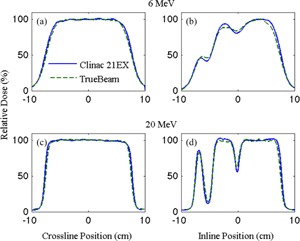
Film measured crossline and inline profiles for 6 and 20 MeV electrons delivered at 70 cm SSD with a $20\times 20\;{\text{cm}}^{2}$ MLC‐shaped aperture with five closed leaf pairs.

Figure 5 shows crossline ((a) and (c)) and inline profiles ((b) and (d)) taken from film measurements of Clinac 21EX and TrueBeam fields shaped using the MLC aperture shown in Fig. 1. Both sets of profiles have been taken 3.5 cm off the central axis, as illustrated by the lines (i) and (ii) in Fig. 1, and are normalized at their intersection point (x=3.5 cm,z=3.5 cm). 6 MeV data are taken at 1.5 cm depth, while 20 MeV data are taken at 2.0 cm depth. Away from the penumbra, the mean standard deviation for each profile measurement ranges from 2.7% to 5.2%, while the maximum standard deviation ranges from 4.5% to 9.7%.

Profiles through the open portion of the aperture ((a) and (c)) agree within 1 SD of the measurement. The modulated portions of the inline profiles show differences of up to 10% between the Clinac 21EX and TrueBeam, but these differences fall within 2 SDs of the measurement.

## IV. DISCUSSION

Despite the changes made to its internal design compared to previous generations of Varian linear accelerators, electron fields generated by the TrueBeam have similar depth and profile parameterizations compared to those generated by the Clinac 21EX, whether delivered using conventional electron applicators at 100 cm SSD or with the photon MLC at 70 cm SSD.

At 6 MeV, applicator data are nearly identical between the machines. At electron energies of 9, 12, 16, and 20 MeV, differences in the applicator depth data fall well within 2% or 2 mm, and compared to the Clinac 21EX, the TrueBeam generates flatter applicator‐shaped fields with reduced photon contamination. Differences in profile data fall largely within 3% or 3 mm, but differences of as much as 8% occur in the shoulder region at larger applicator sizes. Beyer^(2)^ attributed similar differences in photon profile shoulder heights to changes in the incident electron source width, while papers by Weinberg et al.^(10)^ and Huang et al.^(11)^ showed the same relationship for electrons. These profile differences, therefore, can be attributed to differences in the electron source and scattering foil design.

For the three energies investigated using MLC‐shaped fields, differences between the Clinac 21EX and TrueBeam are less pronounced than in applicator‐based measurements. Differences in depth dose data fall, largely, within 2% and 2 mm, while differences in profile data fall within 2% and 3 mm. The differences seen in the shoulders of the applicator‐shaped data are more subtle in the MLC‐shaped measurements. Note that the largest MLC‐shaped aperture investigated was $20\times 20\;{\text{cm}}^{2}$, while the largest applicator‐shaped aperture was $25\times 25\;{\text{cm}}^{2}$, and differences in shoulder height appear to increase with field size. Also note that because the Clinac 21EX and TrueBeam accelerators use the same MLC, differences between MLC‐shaped apertures are not due to MLC design.

Comparisons of film measurements support diode measurement findings, showing good agreement between the machines in both profile directions. Profiles through the open portions of the field show differences within 2% away from the penumbra, and all differences fall within 2 SDs of the measurements. Good alignment in the modulated regions of the inline profiles is indicative of good MLC alignment between the machines perpendicular to the direction of leaf motion.

The electron PDDs and profiles produced by the Clinac 21EX and TrueBeam linear accelerators are very similar, and essentially identical for 6 MeV electrons at the field sizes evaluated in this study. Given this comparison, it is expected that Clinac 21EX beam models may be adapted in order to simulate electron fields that are dosimetrically equivalent to those generated by the TrueBeam.

## V. CONCLUSIONS

MLC‐ and applicator‐based depth and profile measurements of Clinac 21EX and TrueBeam electron fields are very similar and, at 6 MeV, the fields are nearly indistinguishable. The differences that do exist are most pronounced in large applicator‐shaped measurements. Differences in the field widths and penumbras in MLC‐shaped fields and in dose magnitudes in the modulated regions of the film data are within measurement and setup uncertainty, and the excellent spatial match in film‐measured dose shows good alignment of the MLC between the machines. Given the similarities in electron fields, it is reasonable to suggest that source models of a Clinac 21EX linear accelerator may be used as a starting point in order to simulate electron fields that are dosimetrically equivalent to those generated by a TrueBeam accelerator.

## References

[1] Chang Z , Wu Q , Adamson J , et al. Commissioning and dosimetric characteristics of TrueBeam system: composite data of three TrueBeam machines. Med Phys. 2012;39(11):6981–7018. 23127092 10.1118/1.4762682

[2] Beyer GP . Commissioning measurements for photon beam data on three TrueBeam linear accelerators, and comparison with Trilogy and Clinac 2100 linear accelerators. J Appl Clin Med Phys. 2013;14(1):273–88. Retrieved October 22, 2014 from http://www.medphys.org 10.1120/jacmp.v14i1.4077 PMC5714054 23318395

[3] Glide‐Hurst C , Bellon M , Foster R , et al. Commissioning of the Varian TrueBeam linear accelerator: a multi‐institutional study. Med Phys. 2013;40(3):031719. 23464314 10.1118/1.4790563

[4] Rodrigues A , Sawkey DL , Yin F‐F , Wu Q . A Monte Carlo simulation framework for electron beam dose calculations using Varian phase space files for TrueBeam Linacs. Med Phys. 2015;42(5):2389–403. 25979034 10.1118/1.4916896

[5] Salguero FJ , Palma B , Arráns R , Rosello J , Leal A . Modulated electron radiotherapy treatment planning using a photon multileaf collimator for post‐mastectomized chest walls. Radiother Oncol. 2009;93(3):625–32. 19758721 10.1016/j.radonc.2009.08.021

[6] Arjomandy B , Tailor R , Anand A , et al. Energy dependence and dose response of Gafchromic EBT2 film over a wide range of photon, electron, and proton beam energies. Med Phys. 2010;37(5):1942–47. 20527528 10.1118/1.3373523

[7] Chan E , Lydon J , Kron T . On the use of Gafchromic EBT3 films for validating a commercial electron Monte Carlo dose calculation algorithm. Phys Med Biol. 2015;60(5):2091–102. 25674999 10.1088/0031-9155/60/5/2091

[8] Reinhardt S , Hillbrand M , Wilkens JJ , Assmann W . Comparison of Gafchromic EBT2 and EBT3 films for clinical photon and proton beams. Med Phys. 2012;39(8):5257–62. 22894450 10.1118/1.4737890

[9] Garcia LIR and Azorin JFP . Improving the calibration of radiochromic films by the use of uncertainties in optical density and dose. Med Phys. 2013;40(7):071726. 23822430 10.1118/1.4811238

[10] Weinberg R , Antolak JA , Starkschall G , Kudchadker RJ , White RA , Hogstrom KR . Influence of source parameters on large‐field electron beam profiles calculated using Monte Carlo methods. Phys Med Biol. 2009;54(1):105–16. 19075360 10.1088/0031-9155/54/1/007

[11] Huang VW , Seuntjens J , Devic S , Verhaegen F . Experimental determination of electron source parameters for accurate Monte Carlo calculation of large field electron therapy. Phys Med Biol. 2005;50(5):779–86. 15798254 10.1088/0031-9155/50/5/004

